# Enteroviruses isolated from herpangina and hand-foot-and-mouth disease in Korean children

**DOI:** 10.1186/1743-422X-9-205

**Published:** 2012-09-17

**Authors:** KwiSung Park, BaeckHee Lee, KyoungAh Baek, DooSung Cheon, SangGu Yeo, JoonSoo Park, JaeWan Soh, HaeKyung Cheon, KyungAh Yoon, YoungJin Choi

**Affiliations:** 1 Chungcheongnam-Do Institute of Health and Environmental Research, Daejeon, South Korea; 2 JeongGene Pediatrics, Sejong, South Korea; 3 Division of Vaccine Research, Center for Infectious Diseases, National Institute of Health, Korea Center for Disease Control and Prevention, Osong, South Korea; 4 Department of Pediatrics, College of Medicine, Soonchunhyang University, Cheonan, South Korea; 5 Department of Orthopedic Surgery, College of Medicine, Soonchunhyang University, Cheonan, South Korea; 6 Department of Radiological Science, Baekseok Culture University, Cheonan, South Korea; 7 Department of Clinical Pathology, Daejeon Health Sciences College, Daejeon, South Korea; 8 Departments of Laboratory Medicine, College of Medicine, Soonchunhyang University, Cheonan, South Korea

**Keywords:** Herpangina, Hand-foot-and-mouth disea1se, Enteroviruses

## Abstract

Hand-foot-and-mouth disease (HFMD) and herpangina are commonly prevalent illness in young children. They are similarly characterized by lesions on the skin and oral mucosa. Both diseases are associated with various enterovirus serotypes. In this study, enteroviruses from patients with these diseases in Korea in 2009 were isolated and analyzed. Demographic data for patients with HFMD and herpangina were compared and all enterovirus isolates were amplified in the VP1 region by reverse transcription-polymerase chain reaction and sequenced. Among the enterovirus isolates, prevalent agents were coxsackievirus A16 in HFMD and coxsackievirus A5 in herpangina. More prevalent months for HFMD were June (69.2%) and May (11.5%), and June (40.0%) and July (24.0%) for herpangina. Age prevalence of HFMD patients with enterovirus infection was 1 year (23.1%), 4 years (19.2%), and over 5 years (19.2%). However, the dominant age group of herpangina patients with enterovirus infection was 1 year (48.0%) followed by 2 years (28.0%). Comparison of pairwise VP1 nucleotide sequence alignment of all isolates within the same serotypes revealed high intra-type variation of CVA2 isolates (84.6–99.3% nucleotide identity). HFMD and herpangina showed differences in demographic data and serotypes of isolated enteroviruses, but there was no notable difference in amino acid sequences by clinical syndromes in multiple comparison of the partial VP1 gene sequence.

## Introduction

Hand-foot-and-mouth disease (HFMD) and herpangina, which commonly affect young children, are enterovirus infections causing a variety of exanthems. HFMD is a self-limiting exanthematous eruption characterized by vesicles in the oral cavity, mainly in the buccal mucosa and tongue, and peripherally distributed cutaneous lesions on the hands and feet. Herpangina produces multiple oral ulcers affecting predominantly the posterior part of the oral cavity only [1-3]. These diseases are associated with different strains of enteroviruses, such as coxsackievirus A (CVA) 2, 5, 6, 10, 16; coxsackievirus B (CVB) 1, 2, 5; and enterovirus (EV) 71 [4-8].

Human enterovirus (HEV) genera containing the CVA, CVB, echovirus (ECV), and EV serotypes are transmitted mainly via the fecal-oral route and by contact with throat discharges or fluid from blisters [9]. Generally, HEV outbreaks peak during the summer and early fall, and various serotypes are often associated with a single outbreak [10]. Since 1993, when nationwide surveillance began in Korea, there have been reports of summer outbreaks of enteroviruses caused by ECV 5, 6, 7, 9, 13, 18, and 30; CVA 24; CVB 3 and 5; and EV 71 [11,12]. Especially, outbreaks of HFMD and herpangina caused by HEV infection were reported in 2009 in Korea [13,14].

Diagnosis of HEV infections is based on amplification of a highly conserved 5’ non-coding region (NCR) that is widely-targeted in diagnostic procedures [15,16]. In addition to traditional virological methods to serotype HEV, reverse transcription-polymerase chain reaction (RT-PCR) based on amplification of the VP1 region have been recently developed [17-19]. Because the VP1 region is one of the main exposed regions of the viral capsid and has been suggested to include a serotype specific antigenic neutralization site, the BC loop in this region has been implicated with viral antigenicity, and substitutions resulting in conformational changes in this region are believed to play a role in host adaptation for HEVs [20,21].

In this study, to compare epidemic patterns of HFMD and herpangina, specimens from patients with HFMD and herpangina disease were collected and analyzed along with demographic data. Molecular detection by 5’ NCR RT-PCR and sequencing of the VP1 region of HEVs were carried out.

## Materials and methods

### Diagnostic definitions

Herpangina was defined as the presence of oral ulcers on the anterior tonsillar pillars, soft palate, buccal mucosa, or uvula. Patients with HFMD had oral ulcers on the tongue or the buccal mucosa, and vesicular rashes over the palms, soles, knees, or buttocks [22]. This study was conducted in accordance with ethical principles as formulated in the World Medical Association Declaration of Helsinki and approved by the institutional review board (IRB No. 2012–48) of the Ethical Committee of Soonchunhyang University Cheonan Hospital. Additionally, informed consent was obtained from the parents on the patients’ behalf who participated in the study and the parents of participants also gave their consent to publish the data.

### Detection of enterovirus and molecular typing

Stool specimens from 29 HFMD and 32 herpangina patients were collected from collaborative JeongGene Pediatrics hospital in Chungnam province, Korea during 2009. The testing algorithm for detection and molecular typing of HEV has been previously described [12]. Viral RNA was extracted from the supernatant of infected cells using magnetic beads (Toyobo, Osaka, Japan). Extracted RNA was dissolved in 50 μL of nuclease-free water and stored at −70°C until used for RT-PCR.

HEVs were assayed for in clinical samples using a AccuPower® Enterovirus Real-Time RT-PCR Kit (Bioneer, Daejeon, Korea) based on 5’ NCR of highly conserved region in the HEV genome, according to the manufacturer’s instructions [23]. If the sample was positive, it was performed semi-nested PCR in VP1 coding region for molecular typing. Semi-nested PCR condition and primer sequences amplifying the VP1 coding region were described previously [18]. In the initial PCR, a 50 μL reaction mix containing 0.2 μM of primers 224 and 222, 2 U of Taq DNA polymerase (Promega, Madison, WI), 100 μM concentrations of mixture of dNTPs, and 2 μM MgCl2 was amplified by 40 cycles of 95°C for 30 sec, 42°C for 30 sec, and 60°C for 45 s. One microliter of the first PCR product was added to a second PCR for semi-nested amplification. Fifty microliters of a reaction mix containing 0.2 μM of primers AN89 and AN88, 2.5 U of *Taq* DNA polymerase (Promega), 100 μM concentrations of a mixture of dNTPs, and 2 μM MgCl2 was incubated at 95°C for 6 min prior to 40 amplification cycles of 95°C for 30 s, 60°C for 20 s, and 72°C for 15 s. PCR products were purified using the QIA quick PCR purification kit (Qiagen, Valencia, CA). Purified DNA was added in a reaction mixture containing 2 μL of Big Dye terminator reaction mix (Applied Biosystems, Foster City, CA) and 2 pmoles of AN88 and AN89 primers. Sequencing reactions were subjected to initial denaturation at 96°C for 1 min and 25 cycles consisting of 96°C for 10 s, 50°C for 5 s, and 60°C for 4 min in a Gene Amp PCR system 2700 (Applied Biosystems, Foster City, CA). The products were purified by precipitation with 100% cold ethanol and 3 M sodium-acetate (pH 5.8), and then loaded on a model 3100 automated genetic analyzer (Applied Biosystems). The molecular type of each isolates was determined by the serotype of the highest scoring strain in Genbank using the Basic Local Alignment Search Tool (BLAST); that is, the sequence of the HEV strain that gave the highest nucleotide similarity value with the query sequence [24].

### Sequence analysis

Sequence analysis was carried out for five strains of CVA 2, 16 strains of CVA 5, 18 strains of CVA 16, two strains of CVB 1, and six strains of EV71 (Table 1). Nucleotide and deduced amino acid sequences of all HEV isolates were compared with the reference strains using CLUSTAL W (version 1.81) and Megalign (DNASTAR) [25]. The phylogenetic relationships among the VP1 sequences of each virus isolate were inferred using MEGA software v. 5.05. Maximum Composite Likelihood was used as the substitution method, while the neighbor-joining method was used to reconstruct the phylogenetic tree [26]. The reliability of the phylogenetic tree was determined by bootstrap re-sampling of 1,000 replicates. 

**Table 1:** Numbers of enterovirus serotype isolated from patients with HFMD and herpangina

**Serotypes**	**HFMD**		**Herpangina**	
	**Isolate numbers**	**Percentage of subtotal**	**Isolate numbers**	**Percentage of subtotal**
Coxsakievirus A2	0	-	5	20.0
Coxsakievirus A5	3	11.5	13	52.0
Coxsakievirus A16	16	61.5	2	8.0
Coxsakievirus B1	1	3.8	1	4.0
Enterovirus 71	4	15.4	2	8.0
Untypable	2	7.7	2	8.0
Total	26	100	25	100

### Nucleotide sequence accession numbers

The HEV sequences reported here were deposited in the GenBank sequence database under accession numbers JF773151 to JF773197.

## Results

### Enterovirus detection and molecular typing

Among stool specimens from 29 HFMD and 32 herpangina patients, the approach detected 26 HEV isolates (89.7%) in HFMD and 25 HEV isolates (78.1%) in herpangina. The types in the 26 isolates from HFMD patients were mainly identified as CVA 16 (16 isolates, 61.5%), EV 71 (four isolates, 15.4%), CVA 5 (three isolates, 11.5%). The types in the 25 isolates from herpangina patients were mainly identified as CVA 5 (13 isolates, 52.0%), CVA 2 (five isolates, 20.0%) (Table 1).

### Epidemiological features

**Figure 1:**
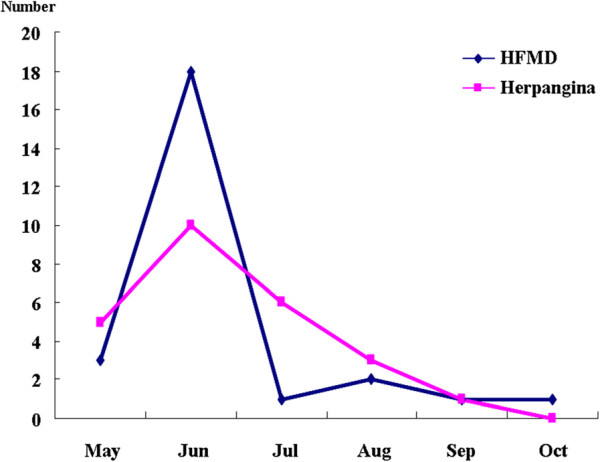
Temporal distribution of enteroviruses isolated from patients with HFMD and herpangina.

**Figure 2:**
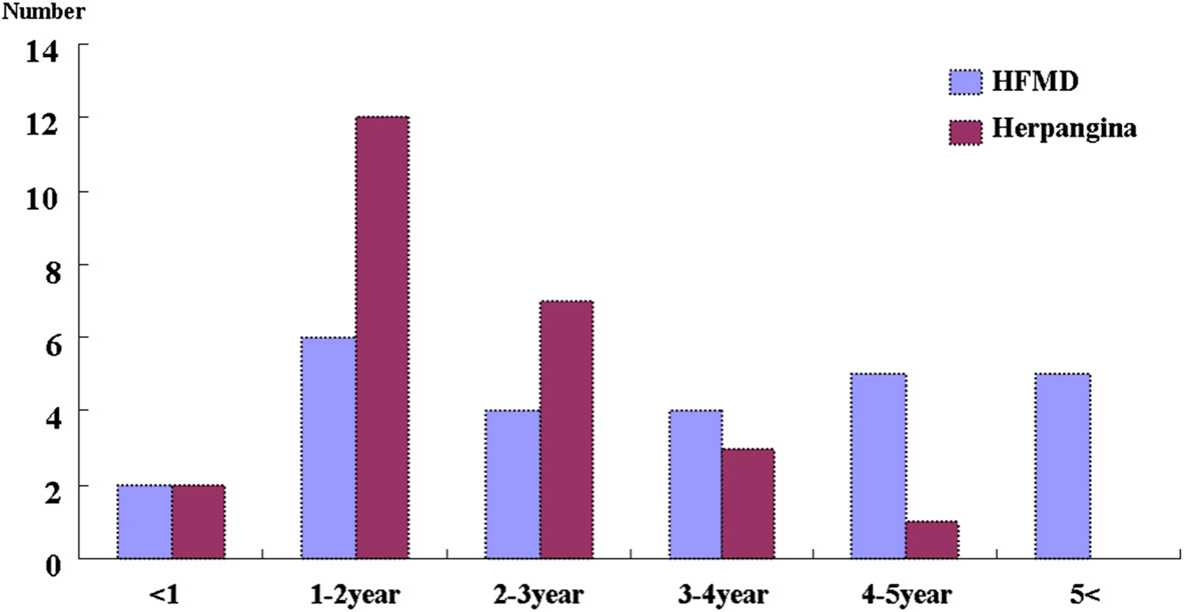
Age distribution of enteroviruses isolated from patients with HFMD and herpangina.

In temporal distribution, HEV positive samples from HFMD patients comprised three isolates (11.5%) in May, 18 isolates (69.2%) in June, one isolate (3.8%) in July, two isolates (7.7%) in August, one isolate (3.8%) in September, and one isolate (3.8%) in October. The HEV positive samples from herpangina patients comprised five isolates (20.0%) in May, 10 isolates (40.0%) in June, six isolates (24.0%) in July, three isolates (12.0%) in August, and one isolate (4.0%) in September (Figures 1). Concerning the age of HFMD patients with enterovirus infection, two isolates (7.7%) were recovered from patients <1-year-of-age, six isolates (23.1%) at 1-year-of-age, four isolates (15.4%) at 2-years-of-age, four isolates (15.4%) at 3-years-of-age, five isolates (19.2%) at 4-years-of-age, and five isolates (19.2%) at >5-years-of-age. Concerning the age of herpangina patients with enterovirus infection, two isolates (8.0%) were recovered at <1-year-of-age, 12 isolates (48.0%) at 1-year-of-age, seven isolates (28.0%) at 2-years-of-age, three isolates (12.0%) at 3-years-of-age, and one isolate (4.0%) at 4-years-of-age (Figures 2).

### Sequence analysis of enteroviruses

**Figure 3:**
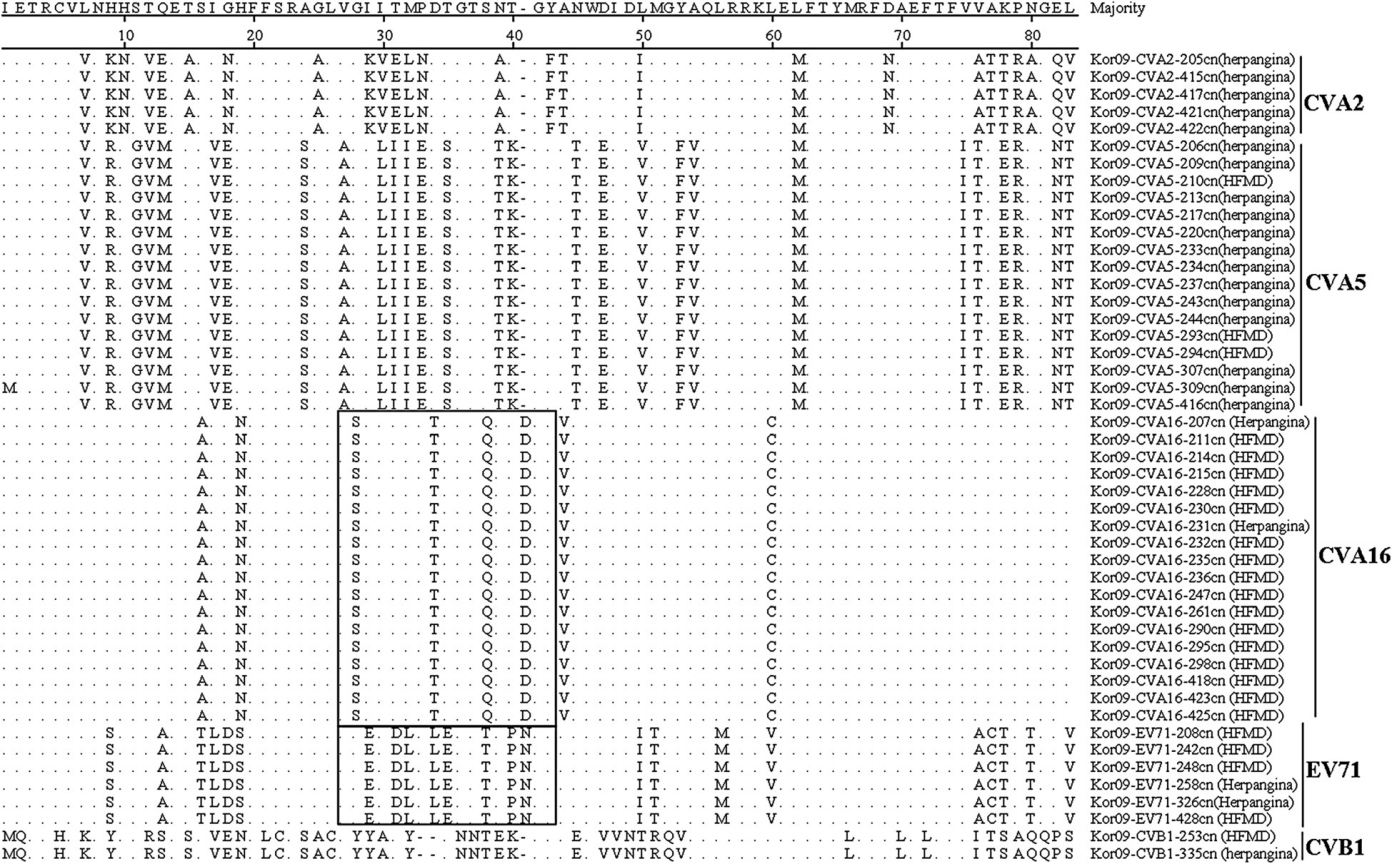
**Comparison of the deduced amino acid sequences of VP1 region of 47 enteroviruses isolated from patients with HFMD and herpangina.** The rectangular parts are BC loop previously reported.

**Figure 4:**
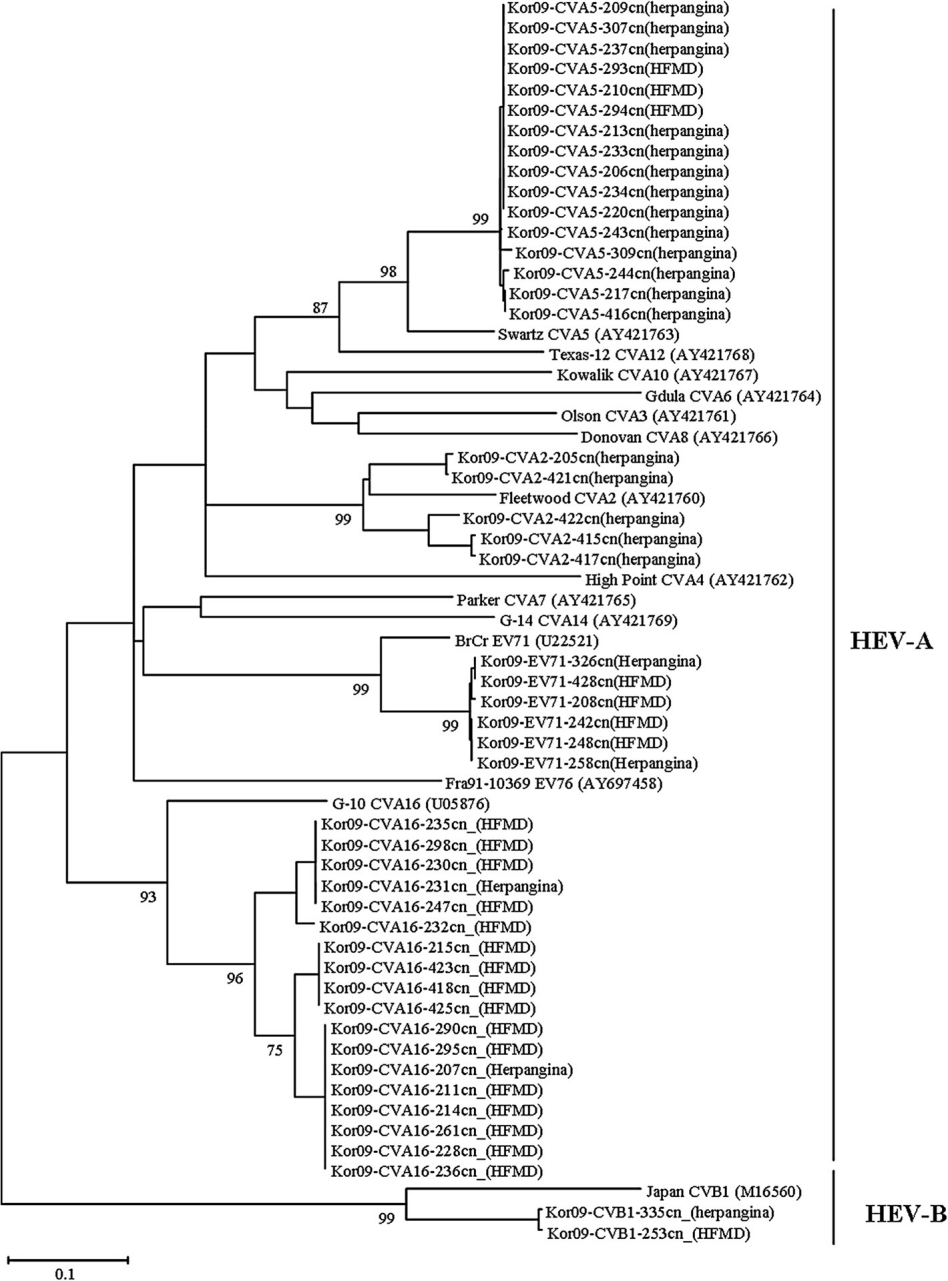
**Phylogenetic analysis based on VP1 region of enteroviruses isolated from patients with HFMD and herpangina.** Nucleotide sequences were analyzed by the neighbor-joining method. The numbers at the branches indicate the bootstrap values for 1,000 replicates.

Based on the VP1 amino acid sequence comparison for all isolates, all sequences represented the same intra-serotype, except for the Kor09-CVA5-309cn (herpangina) isolate, regardless of syndrome (Figures 3). The pairwise VP1 nucleotide sequence alignment of all isolates was compared within the same serotypes; CVA2 isolates displayed 84.6–99.3% nucleotide identity within the intra-type. CVA5, 16, CVB1, and EV71 isolates showed 98.7–100%, 89.6–100%, 99.4%, and 98.9–100% identity, respectively. The phylogenetic relationships of Korean isolates and reference strains are shown in Figures 4.

## Discussion

HFMD and herpangina were epidemic in Korea in 2009 [13,14] and are commonly prevalent illness in young children. They are similarly characterized by lesions on the skin and oral mucosa. Also, these two diseases are associated with various enterovirus serotypes. In HFMD, it has been reported that the major causative HEV types are CVA16 and EV71; these types were also more prevalent serotypes in Korea in 2009 [27,28]. Generally, CVA6 and CVA10 are the more prevalent serotypes in herpangina; however, CVA5 and CVA2 were the prevalent herpangina serotypes in Korea in 2009 [29]. In multiple comparison of partial VP1 gene in the intratypes, no notable variation in amino acid sequences by clinical syndrome was evident. The VP1 region containing the BC loop is associated with viral antigenicity and substitutions of amino acid sequences in this region are believed to be important in host infection of HEVs [20,21,30]. No significant clustered relationship was found according to the clinical syndromes. When the pairwise VP1 nucleotide sequence alignment of all isolates was compared within the same serotypes, CVA2 isolates showed an appreciable variation in nucleotide identity (84.6–99.3%) within the intra-type. Deduced amino acid sequences of all strains were in the same intra-serotype, except for one isolate, regardless of syndromes.

There was no significant difference in age distribution between herpengina (3.74 ± 2.48 years) and HFMD (3.54 ± 2.05 years) in a previous report [31]. But, in this study, age distribution of HEV positive in herpangina patients was relatively lower than in HFMD patients. In the 2009 Korean epidemic, enterovirus infections were most prevalent in July [32]. However, temporal distribution of HEV positive in herpangina and HFMD peaked in June, with herpangina gradually decreased thereafter while HFMD decreased rapidly.

In conclusion, HFMD and herpangina showed differences in demographic data and serotypes of isolated HEVs, but there was no notable difference in amino acid sequences by clinical syndromes in multiple comparison of the partial VP1 gene sequence.

## Competing interests

The authors declare that they have no competing interests.

## Authors’ contributions

KSP, SGY, KAB, JWS, HKC and DSC performed molecular diagnosis and sequence analysis. BHL and JSP contributed to specimen collections and clinical diagnosis. KAY and YJC designed the study and critically revised the manuscript. All of the authors read and approved the final version of the manuscript.

## References

[B1] Bending WA Fleming DM Epidemiological, virological and clinical features of an epidemic of hand, food and mouth disease in England and Wales Commun Dis Rep CDR Rev 1996 6 R81 R86 8664928

[B2] Chang LY Lin TY Huang YC Fulminant neurogenic pulmonary edema with hand, foot and mouth disease Lancet 1998 352 367 368 10.1016/S0140-6736(98)24031-1 9717926

[B3] Rabenau HF Richter M Doerr HW Hand, foot and mouth disease: seroprevalence of Coxsackie A16 and Enterovirus 71 in Germany Med Microbiol Immunol 2009 199 45 51 19941005 10.1007/s00430-009-0133-6

[B4] Flewett TH Warrin RP Clarke SKR Hand, foot and mouth disease associated with Coxsackie A5 virus J Clin Pathol 1963 16 53 55 10.1136/jcp.16.1.53 13945538 PMC480485

[B5] Duff MF Hand-foot-and-mouth syndrome in humans: coxsackie A10 infections in New Zealand Br Med J 1968 2 661 664 10.1136/bmj.2.5606.661 5658411 PMC1991723

[B6] Lindenbaum JE Van Dyck PC Allen RG Hand, foot and mouth disease associated with coksackievirus group B Scand J Infect Dis 1975 7 161 163 1179172 10.3109/inf.1975.7.issue-3.01

[B7] Ishimaru Y Nakano S Yamaoka K Takami S Outbreaks of hand, foot, and mouth disease by enterovirus 71 Arch Dis Child 1980 55 583 588 10.1136/adc.55.8.583 6254449 PMC1627055

[B8] Osterback R Vuorinen T Linna M Susi P Hyypiä T Waris M Coksackievirus A6 and hand, foot and mouth disease, Finland Emerg Infect Dis 2009 15 1485 1488 10.3201/eid1509.090438 19788821 PMC2819858

[B9] Diedrich S Weinbrecht A Schreier E Seroprevalence and molecular epidemiology of enterovirus 71 in Germany Arch Virol 2009 154 1139 1142 10.1007/s00705-009-0413-x 19506798

[B10] Jacques J Moret H Minette D Leveque N Jovenin N Deslee G Lebargy F Motte J Andreoletti L Epidemiological, molecular, and clinical features of enterovirus respiratory infections in French children between 1999 and 2005 J Clin Microbiol 2008 46 206 213 10.1128/JCM.01414-07 18003804 PMC2224256

[B11] Jee YM Cheon DS Choi WY Ahn JB Kim KS Chung YS Lee JW Lee KB Noh HS Park KS Lee SH Kim SH Cho KS Kim ES Jung JK Yoon JD Cho HW Updates on enterovirus surveillance in Korea Inf Chemotherapy 2004 36 294 303

[B12] Baek K Park K Jung E Chung E Park J Choi H Baek S Jee Y Cheon D Ahn G Molecular and epidemiological characterization of enteroviruses isolated in Chungnam, Korea from 2005 to 2006 J Microbiol Biotechnol 2009 19 1055 1064 10.4014/jmb.0810.584 19809266

[B13] Choi CS Choi YJ Choi UY Han JW Jeong DC Kim HH Kim JH Kang JH Clinical manifestations of CNS infections caused by enterovirus type 71 Korean J Pediatr 2011 54 11 16 10.3345/kjp.2011.54.1.11 21359055 PMC3040360

[B14] Kim JH Kim SJ Cheon DS Hand-Foot-Mouth Disease Related to Enterovirus 71 J Korean Med Assoc 2009 52 886 894 10.5124/jkma.2009.52.9.886

[B15] Romero JR Reverse-transcription polymerase chain reaction detection of the enteroviruses Arch Pathol Lab Med 1999 123 1161 1169 10583920 10.5858/1999-123-1161-RTPCRD

[B16] Thoelen I Moes E Lemey P Mostmans S Wollants E Lindberg AM Vandamme AM Van Ranst M Analysis of the serotype and genotype correlation of VP1 and the 5' noncoding region in an epidemiological survey of the human enterovirus B species J Clin Microbiol 2004 42 963 971 10.1128/JCM.42.3.963-971.2004 15004039 PMC356875

[B17] Oberste MS Maher K Kilpatrick DR Pallansch MA Molecular evolution of the human enteroviruses: Correlation of serotype with VP1 sequence and application to picornavirus classification J Virol 1999 73 1941 1948 9971773 10.1128/jvi.73.3.1941-1948.1999 PMC104435

[B18] Nix WA Oberste MS Pallansch MA Sensitive, seminested PCR amplification of VP1 sequences for direct identification of all enterovirus serotypes from original clinical specimens J Clin Microbiol 2006 44 2698 2704 10.1128/JCM.00542-06 16891480 PMC1594621

[B19] Oberste MS Maher K Williams AJ Dybdahl-Sissoko N Brown BA Gookin MS Peñaranda S Mishrik N Uddin M Pallansch MA Species-specific RT-PCR amplification of human enteroviruses: A tool for rapid species identification of uncharacterized enteroviruses J Gen Virol 2006 87 119 128 10.1099/vir.0.81179-0 16361424

[B20] Stirk HJ Thornton JM The BC loop in poliovirus coat protein VP1: An ideal acceptor site for major insertions Protein Eng 1994 7 47 56 10.1093/protein/7.1.47 7511245

[B21] Norder H Bjerregaard L Magnius L Lina B Aymard M Chomel JJ Sequencing of ‘untypable’ enteroviruses reveals two new types, EV-77 and EV-78, within human enterovirus type B and substitutions in the BC loop of the VP1 protein for known types J Gen Virol 2003 84 827 836 10.1099/vir.0.18647-0 12655083

[B22] Chen SP Huang YC Li WC Chiu CH Huang CG Tsao KC Lin TY Comparison of clinical features between coxsackievirus A2 and enterovirus 71 during the enterovirus outbreak in Taiwan, 2008: a children's hospital experience J Microbiol Immunol Infect 2010 43 99 104 10.1016/S1684-1182(10)60016-3 20457425

[B23] Zoll GJ Melchers WJ Kopecka H Jambroes G Van der Poel HJ Galama JM General primer-mediated polymerase chain reaction for detection of enteroviruses: Application for diagnostic routine and persistent infections J Clin Microbiol 1992 30 160 165 1370845 10.1128/jcm.30.1.160-165.1992 PMC265013

[B24] Oberste MS Maher K Flemister MR Marchetti G Kilpatrick DR Pallansch MA Comparison of classic and molecular approaches for the identification of untypeable enteroviruses J Clin Microbiol 2000 38 1170 1174 10699015 10.1128/jcm.38.3.1170-1174.2000 PMC86366

[B25] Thompson JD Higgins DG Gibson TJ Clustal W: Improving the sensitivity of progressive multiple sequence alignment through sequence weighting, position-specific gap penalties and weight matrix choice Nucl Acids Res 1994 22 4673 4680 10.1093/nar/22.22.4673 7984417 PMC308517

[B26] Tamura K Dudley J Nei M Kumar S MEGA4: Molecular Evolutionary Genetics Analysis (MEGA) software version 4.0 Mol Biol Evol 2007 8 1596 1599 17488738 10.1093/molbev/msm092

[B27] Yamashita T Ito M Taniguchi A Sakae K Prevalence of coxsackievirus A5, A6, and A10 in patients with herpangina in Aichi Prefecture, 2005 Jpn J Infect Dis 2005 58 390 391 16377876

[B28] Zong W He Y Yu S Yang H Xian H Liao Y Hu G Molecular phylogeny of Coxsackievirus A16 in Shenzhen, China, from 2005 to 2009 J Clin Microbiol 2011 49 1659 1661 10.1128/JCM.00010-11 21325543 PMC3122795

[B29] Iwai M Masaki A Hasegawa S Obara M Horimoto E Nakamura K Tanaka Y Endo K Tanaka K Ueda J Shiraki K Kurata T Takizawa T Genetic changes of coxsackievirus A16 and enterovirus 71 isolated from hand, foot, and mouth disease patients in Toyama, Japan between 1981 and 2007 Jpn J Infect Dis 2009 62 254 259 19628900

[B30] Lee ST Ki CS Lee NY Molecular characterization of enteroviruses isolated from patients with aseptic meningitis in Korea, 2005 Arch Virol 2007 152 963 970 10.1007/s00705-006-0901-1 17238012

[B31] Lee MH Huang LM Wong WW Wu TZ Chiu TF Chang LY Molecular diagnosis and clinical presentations of enteroviral infections in Taipei during the 2008 epidemic J Microbiol Immunol Infect 2011 44 178 183 10.1016/j.jmii.2011.01.018 21524611

[B32] Baek K Yeo S Lee B Park K Song J Yu J Rheem I Kim J Hwang S Choi Y Cheon D Park J Epidemics of enterovirus infection in Chungnam Korea, 2008 and 2009 Virol J 2011 8 297 10.1186/1743-422X-8-297 21668960 PMC3130694

